# Sorafenib and dacarbazine as first-line therapy for advanced melanoma: phase I and open-label phase II studies

**DOI:** 10.1038/bjc.2011.257

**Published:** 2011-07-12

**Authors:** T Eisen, R Marais, A Affolter, P Lorigan, C Robert, P Corrie, C Ottensmeier, C Chevreau, D Chao, P D Nathan, T Jouary, M Harries, S Negrier, E Montegriffo, T Ahmad, I Gibbens, M G James, U P Strauss, S Prendergast, M E Gore

**Affiliations:** 1 Department of Oncology (R4), Cambridge Biomedical Research Centre, Addenbrooke's Hospital, Hills Road, Cambridge CB2 0QQ, UK; 2 Division of Cancer Biology, Institute of Cancer Research, London SW3 6JB, UK; 3 Department of Medical Oncology, Christie Hospital, Withington, Manchester M20 4BX, UK; 4 Department of Medicine, Institut Gustave-Roussy, Villejuif 94805, France; 5 Cancer Sciences Division, Southampton University Hospitals, Southampton SO16 6YD, UK; 6 Department of Medical Oncology, Institut Claudius Regaud, Toulouse Cedex 31052, France; 7 Cancer Services Division, The Royal Free Hospital, London NW3 2QG, UK; 8 Cancer Services Division, Mt Vernon Cancer Centre, Northwood, Middlesex HA6 2RN, UK; 9 Department of Dermatology, Hop̂ital Saint André, Bordeaux Cedex 33075, France; 10 Cancer Services Division Guy's and St Thomas’ Hospital, London SE1 7EH, UK; 11 Cancérologie Médicale, Centre Léon Bérard, Lyon Cedex 39373, France; 12 Bayer plc, Strawberry Hill, Newbury, Berkshire RG14 1JA, UK; 13 Department of Medicine, Royal Marsden Hospital, London SW3 6JJ, UK; 14 Bayer Schering Pharma, Leverkusen 51368, Germany

**Keywords:** melanoma, sorafenib, dacarbazine, combination therapy, biomarker

## Abstract

**Method:**

The safety of oral sorafenib up to a maximum protocol-specified dose combined with dacarbazine in patients with metastatic, histologically confirmed melanoma was investigated in a phase I dose-escalation study and the activity of the combination was explored in an open-label phase II study.

**Results:**

In the phase I study, three patients were treated with sorafenib 200 mg twice daily (b.i.d.) plus 1000 mg m^−2^ dacarbazine on day 1 of a 21-day cycle and 15 patients had the sorafenib dose escalated to 400 mg b.i.d. without reaching the maximum tolerated dose of the combination. In the phase II study (*n*=83), the overall response rate was 12% (95% CI: 6, 21): one complete and nine partial, with median response duration of 46.7 weeks. Stable disease was the best response in 37% median duration was 13.3 weeks. Median overall survival (OS) was 37.0 weeks (95% CI: 33.9, 46.0).

**Conclusion:**

Oral sorafenib combined with dacarbazine had acceptable toxicity and some antineoplastic activity against metastatic melanoma.

The incidence of melanoma worldwide has been on the rise for at least 30 years, with rates as high as 3–5% per year in high-risk populations (Danson and Lorigan, 2005; MacKie *et al*, 2009; American Cancer Society, 2010). Mortality rates associated with melanoma have been decreasing in the younger patient population by 2% to 3% per year since 1990, but increasing in the older population by <1% per year (American Cancer Society, 2010). The prognosis for patients with metastatic disease is poor; the highest quoted 5-year survival rate for patients with stage IV melanoma is 18% with a median survival of 8 months (Balch *et al*, 2001; Gimotty *et al*, 2005; Thompson *et al*, 2005). Although dacarbazine is the standard of care for advanced disease and has an acceptable toxicity profile, it has a low objective response rate of <20% with a median progression-free survival (PFS) of <2 months and no proven survival benefit (Middleton *et al*, 2000; Eggermont and Kirkwood, 2004; Bedikian *et al*, 2006; Flaherty, 2006). In recent clinical studies, more promising outcomes have been observed with new systemic therapies that target immunoregulatory molecules or have highly selective inhibition of mutant pathway-signalling molecules (Hodi *et al*, 2010; Flaherty *et al*, 2010a).

The Raf/MEK/ERK pathway regulates cell proliferation, differentiation, and survival (Roberts and Der, 2007). Activating mutations in the components of this pathway are implicated in 60–90% of melanomas. Mutations in *NRAS* and *BRAF* are present in ∼20% and >50% of melanomas, respectively (Davies *et al*, 2002; Akslen *et al*, 2005). *In vitro* studies have demonstrated that sorafenib is a selective multikinase inhibitor targeting Raf kinases and receptor tyrosine kinases, including platelet-derived growth factor and vascular endothelial growth factor (VEGF) receptors, and had antitumour activity in preclinical models of melanoma (Karasarides *et al*, 2004; Sharma *et al*, 2005; Wilhelm *et al*, 2008). Although sorafenib was not effective as a single agent in advanced melanoma (Eisen *et al*, 2006), it was thought worthwhile to explore the utility of sorafenib in combination with chemotherapeutic agents in the treatment of patients with advanced disease, especially given the absence of efficacious treatment options.

The present paper reports the results from two studies investigating sorafenib–dacarbazine combination therapy in patients with advanced melanoma. The phase I study was designed to explore the safety of sorafenib up to a maximum protocol-specified dose in combination with dacarbazine, as well as to provide insight into the efficacy of the combination. Promising results in the phase I study led to the initiation of the open-label single treatment group phase II study, which was designed to further explore the efficacy and safety of this regimen.

## Patients and methods

### Patient selection

In both studies, patients with metastatic, histologically confirmed melanoma were eligible for inclusion. The main inclusion criteria were: age ⩾18 years; Eastern Cooperative Oncology Group (ECOG) performance status ⩽1; life expectancy ⩾12 weeks; and adequate bone marrow, liver, and renal function. The main exclusion criteria were: primary ocular or mucosal melanoma; previous or concurrent cancer distinct from the cancer evaluated in this trial; clinically evident congestive heart failure; cardiac arrhythmia; coronary artery disease; ischaemia; active, clinically serious infections; chronic hepatitis B or C; and active metastatic brain or leptomeningeal tumours. Previous chemotherapy, radiotherapy ⩽3 weeks, surgery ⩽4 weeks before the first dose of study drug (major surgery in phase II study), or treatment with inhibitors of the *RAS* pathway (including trastuzumab, EGFR inhibitors, farnesyl transferase inhibitors, or MEK inhibitors), or with a VEGF-targeting drug was prohibited. Previous immunotherapy or cytokine, biologic, or vaccine therapy was permitted. Before participation in the phase II study, a 4-week recovery was required after previous immunotherapy, cytokine, and biologic administration, and 3 months after previous vaccine therapy.

### Study design

The phase I single-centre, open-label study was conducted in the United Kingdom between April 2004 and May 2005. The study had two phases: the dose-escalation phase (cohorts 1 and 2) and the expansion phase (expansion cohort 2). Patients in all cohorts received a 1-h intravenous (i.v.) infusion of 1000 mg m^−2^ dacarbazine on day 1 of a 21-day cycle, with this dose maintained throughout the study. Cohort 1 (three patients) received 200 mg oral sorafenib twice daily (b.i.d.) from days 1 to 21. If safe and tolerated, the dosage of sorafenib was to be increased to 400 mg b.i.d. in cohort 2 (three patients). If safety data warranted, cohort 1 could be expanded to six patients and cohort 2 expanded to nine patients. When the MTD of sorafenib was established, or the maximum allowed full dose (400 mg b.i.d.) was reached, a maximum of up to 18 patients receiving combination therapy at this dose could be enroled into an expansion cohort (expansion cohort 2).

The phase II two-stage, open-label, uncontrolled study was conducted at eight sites in the United Kingdom and four sites in France between April 2005 and June 2008. Based on tumour activity observed in stage I, patients could enrol in stage II. During each stage, patients received a 1-h i.v. infusion of 1000 mg m^−2^ dacarbazine on day 1 of a 21-day cycle and 400 mg b.i.d. sorafenib continuously.

Sorafenib tablets were supplied by Bayer plc (Newbury, Berkshire, UK). Dacarbazine was supplied by the study-site pharmacy. The studies were conducted in accordance with the Declaration of Helsinki. Local institutional review boards or independent ethics committees approved the protocols. Written informed consent was obtained from all patients.

### Study outcomes

The MTD of sorafenib in combination with 1000 mg m^−2^ dacarbazine was investigated in the phase I study. The MTD was defined as the maximum dose that could be given to six patients, with not more than two patients experiencing a dose-limiting toxicity (DLT). The DLT was defined as treatment-related occurrence of grade 4 neutropenia, decrease in platelet count to <25 000 *μ*l^−1^, or grade 3 or 4 nonhaematological toxicity as described by the National Cancer Institute Common Terminology Criteria for Adverse Events, version 3.0 (NCI CTCAE v3). The DLT assessments were made in the first cycle of chemotherapy.

#### Safety

For both phase I and II studies, patients who received at least one dose of treatment and who were assessed for safety at least once after treatment were included in safety analyses. The safety profile was based on reported adverse events (AEs), physical examinations, clinical laboratory tests, vital sign measurements, and electrocardiograms. Safety assessments occurred on days 1, 8, and 15 (cycles 1 and 2), day 1 (subsequent cycles), at end of treatment, and at follow-up (28 days post treatment in phase I and active follow-up visits in phase II). The AEs were classified and graded using the NCI CTCAE v3.

#### Efficacy

In both studies, efficacy results were calculated for patients who completed at least one cycle of treatment and had their disease re-evaluated. Where applicable, patients without a disease re-evaluation were included in the denominator of the efficacy analysis. In phase I, evaluation of tumour response was a secondary objective, with variables including tumour response, overall response duration, and overall survival (OS). For the phase II study, the primary end point was overall tumour response rate (ORR; complete response (CR) plus partial response (PR) rates). Secondary end points included: response duration (first determination of CR or PR to progression), disease control rate (DCR; the sum of CR and PR and stable disease (SD), OS (treatment start to death), TTP (treatment start to progression), and PFS.

In both studies, tumour measurements were made at baseline and every two cycles (6 weeks) up to cycle 8 and every four cycles thereafter (phase II only) using Response Evaluation Criteria in Solid Tumours (RECIST) (Therasse *et al*, 2000). Per RECIST guidelines, CR and PR required confirmation by a scan at ⩾6 weeks; SD required a measurement at ⩾6 weeks.

#### Biomarkers

Tumour and normal (punch) skin biopsy samples collected at baseline were frozen in liquid nitrogen for genomic DNA isolation and analysis of genetic mutations *BRAF*, *NRAS*, *KRAS*, and/or *PI3K* by DNA polymerase chain reaction (PCR) techniques performed at the laboratory of Dr Richard Marais at the Institute of Cancer Research (London, UK). PCR products were purified by gel electrophoresis and sequenced with primers used in the amplification step. Automated dideoxy sequencing was performed using Big-Dye Terminator RR mix (Applied Biosystems, Foster City, CA, USA) and analysed using the Sequencer 4.2.1 program (Gene Codes Corporation, Ann Arbor, MI, USA). DNA genotype analysis included exons 11 and 15 of *BRAF* (phase I and II studies), exons 2 and 3 of *NRAS* and *KRAS* (phase I study), and also exons 9 and 20 of *PI3K* (phase II study).

### Statistical analysis

As the phase I study was primarily a descriptive analysis of safety and tolerability, no formal sample size estimation was performed, with a rule-based design being used (Simon, 2008). A maximum of 30 patients could be enroled, with a maximum of 15 in the dose-escalation cohort, as safety data warranted, and a maximum of 18 patients in the expansion cohort.

Using a Simon two-stage optimal design for the phase II study, the sample size was estimated to be 82 (30 patients in the first stage and 52 in the second) (Simon, 1989). If the number of responses (complete and partial) was less than six (20%) in stage I, it would be concluded that the treatment had insufficient efficacy and the trial would be stopped. Although the results at the end of stage 1 showed only five responses in the first 30 treated subjects, the study was amended to allow enrolment of subjects for stage 2 for the following reasons: (1) studies in renal cell carcinoma have shown sorafenib to be efficacious (PFS) in spite of low response rates (Ratain *et al*, 2006; Escudier *et al*, 2007), and similar results were reported in hepatocellular carcinoma (Llovet *et al*, 2008); (2) the median PFS at the end of stage I of the study was 129 days (4.3 months) compared with historical data of 1.5 to 1.6 months in phase III studies with dacarbazine monotherapy (Middleton *et al*, 2000; Eggermont and Kirkwood, 2004; Bedikian *et al*, 2006); (3) at the end of stage I, 15 (50%) patients had a best response of SD with median duration of 104 days; and (4) the combination treatment had not resulted in unacceptable toxicity by the end of stage I.

If the total number of responses in stages I and II were <18 (22%), it would be concluded that the combination treatment did not provide a greater response than dacarbazine alone. If there were ⩾6 responses (⩾20%) in stage I and at least 18 responses in all (22%), the conclusion would be that the treatment had sufficient efficacy to warrant further evaluation.

## Results

In all, 20 patients enroled in the phase I study; 18 patients were treated; all were eligible for inclusion in safety and efficacy analyses. Two patients were excluded during the screening period because of violation of eligibility criteria (presence of brain metastases and previous chemotherapy). Of the 18 patients who received treatment, 4 (22%) discontinued treatment because of AEs and 14 (70%) owing to disease progression. In the phase II study, 96 patients were enroled; 13 were excluded during screening because of violation of eligibility criteria (brain metastasis, *n*=5; abnormal lab values, *n*=5; no lesion, *n*=1; cardiac arrhythmia, *n*=1; AE, *n*=1). In all, 32 patients were treated in stage I and 51 in stage II; all 83 patients were eligible for inclusion in safety and efficacy analyses. Of these 83 patients, 16 (19%) discontinued treatment because of AEs, 60 patients (72%) owing to disease progression, 2 patients (2%) because of death (1 due to treatment-related haemorrhage and 1 due to thrombosis that was considered to be disease-related), 2 patients (2%) because of withdrawal of consent and 3 patients (4%) because of study termination. The baseline characteristics of patients in both studies are presented in Table 1.

### Maximum tolerated dose of sorafenib

Three patients in cohort 1 received 200 mg sorafenib b.i.d. and did not experience DLTs. One of three patients in cohort 2 (400 mg b.i.d.) experienced dose-limiting grade 3 hand–foot skin reaction. Three additional patients were enroled in cohort 2 and did not experience DLTs. As the maximum allowed dose of sorafenib for this study was 400 mg b.i.d., no further dose escalation was carried out. Nine patients were enroled in expansion cohort 2 and treated with 400 mg b.i.d. sorafenib. No DLTs were experienced by these patients (Supplementary Figure 1). Thus, the sorafenib dose selected for use in combination with dacarbazine in the phase II study was 400 mg b.i.d.

#### Safety

In the phase I study, all patients received ⩾90% of the planned doses of both sorafenib and dacarbazine. In the phase II study, 71 patients (86%) received ⩾90% of the planned dose of dacarbazine and 12 patients (14%) received 70% to <90% the mean dacarbazine dose per cycle was 973 mg m^−2^. Nine patients (11%) received 50% to <70% of the planned dose of sorafenib, 57 patients (69%) received 30% to <50%, and 17 patients (20%) received 10% to <30% the mean sorafenib dose per cycle was 623 mg. Sorafenib dose reductions occurred in 25 patients and interruptions in 61 patients; >95% were because of AEs alone or in combination with other reasons. Further dosing and drug exposure data are presented in Supplementary Tables 1A and B.

Common treatment-related AEs in both studies were predominantly grade 1/2 and included events in blood/bone marrow, gastrointestinal, dermatology/skin, constitutional symptoms, neurological symptoms, and pain. The most frequently occurring treatment-emergent grade 3/4 AEs are listed in Table 2.

In the phase I study, serious treatment-emergent AEs occurred in 11 (61%) patients; events occurring in >1 patient were abnormal haemoglobin and lipase levels, seizures, and tumour pain (2 patients each). Most AEs occurred after the first treatment cycle, and were not considered DLTs.

In the phase II study, serious treatment-emergent AEs occurred in 40 patients (48%) and included abnormal haemoglobin and platelet levels, fatigue, fever, thrombosis/embolism, and abdominal pain. In both studies, 22% of patients (4 in phase I and 18 in phase II) reported serious treatment-emergent AEs that were drug related. One patient in the phase II study experienced a grade 1 radiation recall skin reaction in response to treatment. Thyroid function tests were not routinely performed in these studies. A total of 15 deaths were reported in the phase I study; 5 occurred within 30 days after sorafenib treatment ended (progression of metastatic melanoma, *n*=4; renal failure-metastatic melanoma, *n*=1). In the phase II study, 66 deaths were reported; none occurred during study treatment and 9 occurred within 30 days of the end of study treatment (disease progression, *n*=6; metastatic melanoma, *n*=1; haemorrhage, *n*=1; and thrombosis, *n*=1). One of the deaths (cerebrovascular haemorrhage) was considered by the investigator to be related to study treatment.

#### Efficacy

**Figure 1:**
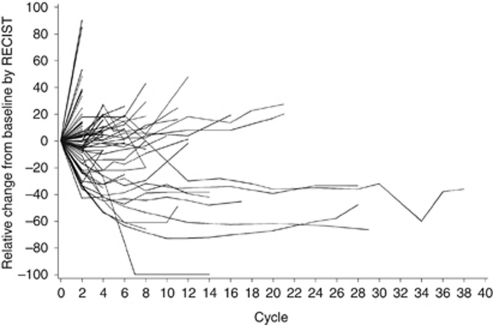
Phase II study: percentage change in tumour burden with time.

**Figure 2:**
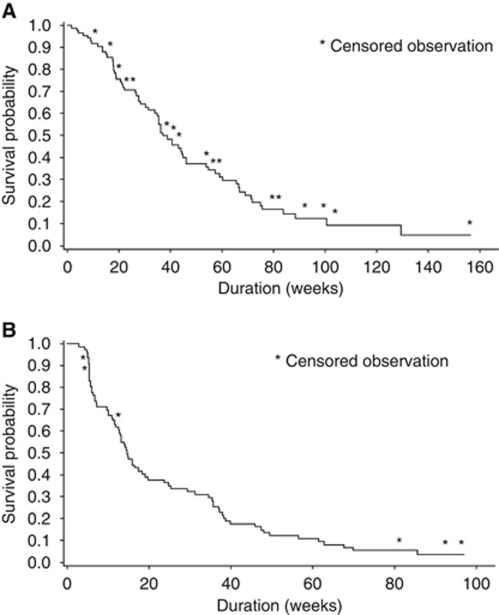
Phase II study Kaplan–Meier plots: (**A**) overall survival and (**B**) time to progression. Data available from 78 subjects for time to progression.

The efficacy data for both studies are presented in Table 3. The change in tumour burden over time for all patients in the phase II study is illustrated in Figure 1 and the largest change per patient is provided in Supplementary Figure 2; Kaplan–Meier plots for OS and TTP are presented in Figures 2A and B, respectively.

In the phase II study, sorafenib combined with dacarbazine was associated with a confirmed, independently assessed ORR of 12% (95% CI: 6, 21), comprising one CR and nine PRs (Table 3). The median duration of response for the CR and PRs was 46.7 weeks (30% censorship) and the median time to response was 48 days. An additional 31 (37%) patients had SD as best response, with a median duration of 13.3 weeks (7% censorship). The median OS was 37.0 weeks (95% CI: 33.9, 46.0). Three patients from the study entered the Sorafenib Long-Term Extension Programme (STEP).

#### Biomarkers

##### Mutation status of *BRAF*, *RAS*, and *PI3K*

In the phase I study, tumour biopsies from nine patients were analysed for mutation status of *BRAF* and *RAS*; normal skin samples were available from seven of the nine patients. Of the nine tumour samples, 5 had activating mutations in exon 15 of *BRAF*; four were V600E *BRAF* mutations and one was a V600K *BRAF* mutation. None of the tumour samples had mutations in exon 11 of *BRAF* or in exons 2 and 3 of *NRAS* and *KRAS*. No *BRAF* or *RAS* mutations were detected in control skin biopsies. There was no apparent correlation between *BRAF* mutation status and response to treatment.

In phase II, 20 tumour samples were obtained to investigate mutation status of *BRAF* and the *PI3K* subunit, *P13KCA.* Three of the samples had V600E mutations in exon 15 of *BRAF*; no mutations in exon 11 of *BRAF* or exons 9 and 20 of *PI3K* were found. The low incidence is likely because of the small sample size. Given that samples from only a minority of patients had mutational analysis, we cannot exclude the possibility that we inadvertently introduced a bias that reduced the chance of sampling patients with a V600E mutation in their tumour. Two of the three patients with V600E mutations had progressive disease as best response; the third was not assessable. All three had shorter OS and two had TTP shorter than the study median (OS: 95 days, 122 days, 26 days; TTP: 50 days, 71 days, not available for one patient).

## Discussion

The current studies were designed to determine the safety and efficacy of the sorafenib/dacarbazine combination in patients with advanced melanoma. The dose-escalation period of the phase I study demonstrated that the regimen was well tolerated. Given our experience with single-agent sorafenib in melanoma (Eisen *et al*, 2006), we wanted to obtain preliminary efficacy data; therefore, the phase I study design included an option for enrolment into the expansion cohort. Based on the activity noted in the phase I study, the phase II clinical trial was initiated.

In the phase II study, the ORR was 12% thus, the study did not meet its predefined primary end point. However, end points such as PFS or TTP that measure disease stabilisation are better suited to measure the activity of sorafenib (Stone *et al*, 2007). Results from a randomised double-blind phase II study published previously also reported the efficacy and safety of the sorafenib/dacarbazine combination in patients with advanced melanoma (McDermott *et al*, 2008). This study did not meet its primary end point of PFS; however, an improvement in PFS with sorafenib plus dacarbazine compared with placebo plus dacarbazine was reported at 6 and 9 months (secondary and tertiary end points in the study). A significant improvement in TTP was observed. In this context, it is interesting to note that the median duration of response in this study was a surprisingly long 46.7 weeks. This might substantiate the findings of the randomised trial by McDermott *et al* (2008).

A comparison of results in different phase II studies is fraught with risk. Nevertheless, some hypotheses can be considered. Given this proviso, lower efficacy was observed in all end points of the present study than in the randomised phase II study. The ORR in the sorafenib plus dacarbazine group of the randomised study was 24% compared with 12% in the current study; median TTP was 21.1 weeks *vs* 15.0 weeks, and median OS values were 45.6 weeks *vs* 37.0 weeks, respectively (McDermott *et al*, 2008). The percentage of patients with AJCC stage IV M1c disease was greater in the present study (80%) compared with that in the randomised study (55%). On the other hand, the percentage of patients with elevated lactate dehydrogenase was notably low in the study reported here, which would be expected to select a relatively lower-risk population. Thus, the differences between the studies cannot be explained by differences in patient characteristics.

In studies of sorafenib in combination with carboplatin (C)/paclitaxel (P), the phase I/II data were promising (Flaherty *et al*, 2008); however, two randomised phase III trials (one of first-line and one of second-line therapy) demonstrated no improvement in the primary end point of PFS for sorafenib plus C/P *vs* C/P alone (Hauschild *et al*, 2009; Flaherty *et al*, 2010b). In the current phase II study as well as in the randomised phase II study (McDermott *et al*, 2008), both conducted in a first-line setting and at similar mean daily sorafenib doses (623.1 mg and 640.5 mg, respectively), the sorafenib/dacarbazine combination demonstrated some evidence of activity regardless of failure of both to meet the predefined objectives. No further studies investigating this combination are currently planned.

The present phase I and II studies demonstrated a clinically acceptable toxicity profile for the sorafenib/dacarbazine combination regimen with dose reductions as per protocol. It should be noted that all patients who continued on treatment required dose reductions to below 70% of the dose planned at study entry, particularly in later cycles. For the most part, the safety profile was comparable with that observed in the phase II randomised study at a similar mean daily sorafenib dose, with the exception that although grade 3/4 CNS haemorrhages were observed in the randomised study, no CNS haemorrhages were reported in the current studies (McDermott *et al*, 2008).

A recent preclinical study demonstrated that, *in vitro*, low concentration of sorafenib promoted MAPK signalling through stabilisation of activating mutant BRAF complexes (Karreth *et al*, 2009). In contrast to sorafenib, recent studies of vemurafenib (PLX4032), a kinase inhibitor that specifically targets mutated BRAF, produced single-agent responses in BRAF-mutant melanoma but did not demonstrate antitumour activity in patients without an activated BRAF mutation (Flaherty *et al*, 2010a). This is consistent with the hypotheses that either sorafenib does not effectively inhibit BRAF in melanoma or that one of the other effects of sorafenib, such as CRAF inhibition, counters any RAF inhibitory effect that is achieved (Karreth *et al*, 2009).

In the current phase II study, mutations in *PI3K* were also investigated, as the PTEN/PI3K/Akt pathway exhibits elevated activity in a large fraction of melanoma tumours. However, no *PI3K* mutations were detected in the samples analysed. The loss of PTEN, which is a more frequent occurrence, was not assessed. No conclusions can be drawn regarding the utility of *BRAF* mutations as biomarkers to predict response because of the small sample size for biomarker analyses in both studies. However, when viewed with similar data from other studies, it seems reasonable to conclude that there is no evidence that *BRAF* status predicts response to sorafenib either as a single agent or in combination with chemotherapy (Eisen *et al*, 2006; Amaravadi *et al*, 2007; Flaherty *et al*, 2008).

Given this lack of correlation, the antitumour activity detected in this trial may have been because of the antiangiogenic properties of sorafenib as an inhibitor of VEGF. In recent trials, other molecules that target angiogenesis receptors have shown potential benefit in melanoma (Fruehauf *et al*, 2008; O’Day *et al*, 2009; Hong *et al* 2010). Furthermore, in an *in vitro* study investigating the potential for continuous low-dose antiangiogenic chemotherapy, sorafenib inhibited the growth of both normal endothelial and melanoma cells (Murray *et al*, 2010).

The results of the two present studies show that the combination of sorafenib and dacarbazine has a manageable toxicity profile and exhibits some evidence of activity. Given the substantial antitumour activity of BRAF inhibitors such as vemurafenib in BRAF-mutant disease, there seems little rationale for pursuing the combination of sorafenib and dacarbazine in that setting; the antitumour activity of sorafenib detected in this study could be further explored in patients without activating BRAF mutations.

## Figures and Tables

**Table 1:** Baseline characteristics of patients

**Characteristic**	**Phase I (*N*=18)**	**Phase II (*N*=83)**
*Sex,* n *(%)*		
Male	12 (67)	50 (60)
Female	6 (33)	33 (40)
		
*Race,* n *(%)*		
White	18 (100)	82 (99)
Asian	0	1 (1)
Age, years, median (range)	55 (36–72)	56 (25–78)
		
*ECOG performance status,* n *(%)*		
0	4 (22)	52 (63)
1	14 (78)	28 (34)
Missing	0	3 (4)
		
*Previous therapy,* n *(%)*		
Radiotherapy	4 (22)	11 (13)
Anticancer surgery	18 (100)	46 (55)
Adjuvant systemic anticancer treatment	5 (28)	18 (22)
		
*Metastatic sites (⩾20% of patients),* n *(%)*		
Lung	16 (89)	53 (64)
Liver	9 (50)	40 (48)
Abdomen	7 (39)	12 (14)
Pelvis	5 (28)	4 (5)
Lymph node	4 (22)	34 (41)
Spleen	4 (22)	5 (6)
		
*Number of tumour sites,* n *(%)*^a^		
1	1 (6)	8 (10)
2	3 (17)	20 (24)
3	4 (22)	16 (19)
⩾4	10 (56)	38 (46)
Missing	0	1 (1)
		
*AJCC stage,* n *(%)*^a^^,^^b^		
IV M1a	0	4 (5)
IV M1b	1 (6)	12 (14)
IV M1c	16 (89)	66 (80)
Missing	1 (6)	1 (1)
		
*Lactate dehydrogenase levels,* n *(%)*		
<10% over ULN	4 (22)	54 (65)
⩾10% over ULN	12 (67)	26 (31)
Missing	2 (11)	3 (4)

Abbreviations: AJCC=American Joint Committee on Cancer; ECOG=Eastern Cooperative Oncology Group; ULN=upper limit of normal.

a Percentages do not add up to 100% due to rounding up of numbers.

b For the phase II study, AJCC stage was supplied separately by the medical expert.

**Table 2:** Treatment-emergent grade 3 or 4 adverse events in ⩾10% of patients

	**Phase I**		**Phase II**	
**Event**	**Cohort 1 (*n*=3) *n* (%)**	**Cohort 2^a^** **(*n*=15)** ***n* (%)**	**Total (*N*=18)** ***n* (%)**	**Total (*N*=83)** ***n* (%)**
*Blood/bone marrow*				
Neutrophils	0	2 (13)	2 (11)	30 (36)
Leukocytes	0	2 (13)	2 (11)	6 (7)
Platelets	0	0	0	18 (22)
				
*Cardiac*				
Hypertension	2 (67)	1 (7)	3 (17)	2 (2)
Fatigue	0	4 (27)	4 (22)	7 (8)
				
*Infection*				
Febrile neutropenia	0	3 (20)	3 (17)	1 (1)
				
*Metabolic/laboratory any event*				
Lipase	1 (33)	2 (13)	3 (17)	2 (2)
				
*Neurology*				
Seizure	1 (33)	0	1 (6)	NR
				
*Pain*				
Tumour pain	0	2 (13)	2 (11)	0
Other	0	2 (13)	2 (11)	NR
Renal failure	0	2 (13)	2 (11)	NR

Abbreviation: NR=not reported.

a Includes cohort 2 and expansion cohort 2.

**Table 3:** Best response, overall survival, and time to progression

	**Phase I**		**Phase II**	
**Efficacy variables**	**Cohort 1 (*n*=3)**	**Cohort 2^a^** **(*n*=15)**	**Total^b^** **(*N*=18)**	**Total** (***N*=83)**
*Best response*				
Patients not evaluable, *n* (%)	0	1 (7)	1 (6)	8 (10)
				
CR				
*n* (%)	0	0	0	1 (1)
95% CI				0, 7
				
PR				
*n* (%)	1 (33)	2 (13)	3 (17)	9 (11)
95% CI	1, 91	2, 41	4, 41	5, 20
				
SD				
*n* (%)	2 (67)	9 (60)	11 (61)	31 (37)
95% CI	9, 99	32, 84	36, 83	27, 49
				
Progressive disease				
*n* (%)	0	3 (20)	3 (17)	34 (41)
95% CI		4, 48	4, 41	30, 52
				
*Overall best response rate (CR+PR)*				
*n* (%)	1 (33)	2 (13)	3 (17)	10 (12)
95% CI	1, 91	2, 41	4, 41	6, 21
				
*Overall survival*				
Patients not evaluable, *n* (%)	0	0	0	0
Censorship rate, %	0	20	17	20
Median, weeks	30.1	23.1	26.1	37.0
95% CI	26.1, 35.6	17.0, 32.6	17.3, 31.7	33.9, 46.0
				
*Time to progression*				
Patients not evaluable, *n* (%)	0	1 (7)	1 (6)	5 (6)
Censorship rate, %	0	29	24	8
Median, weeks	22.7	13.0	13.0	14.6
95% CI	11.3, 33.6	11.3, 19.6	11.3, 19.6	12.6, 19.9

Abbreviations: CI=confidence interval; CR=complete response; PR=partial response; SD=stable disease.

a Includes cohort 2 and expansion cohort 2.

b Response rate (%) and 95% CI based on intent-to-treat patients.
