# Supplementary material for: Sorafenib and dacarbazine as first-line therapy for advanced melanoma: phase I and open-label phase II studies
Source: Br J Cancer. 2011 Jul 12;105(3):353–9. doi: 10.1038/bjc.2011.257 (PMC3172912; doi:10.1038/bjc.2011.257)
Supplement: Supplementary Figures 1 and 2 [file bjc2011257x1.doc]

**Supplemental Figure 1** Phase I study: determination of recommended phase II dose of sorafenib in combination with 1000 mg m^–2^ dacarbazine for treatment of advanced melanoma.

**
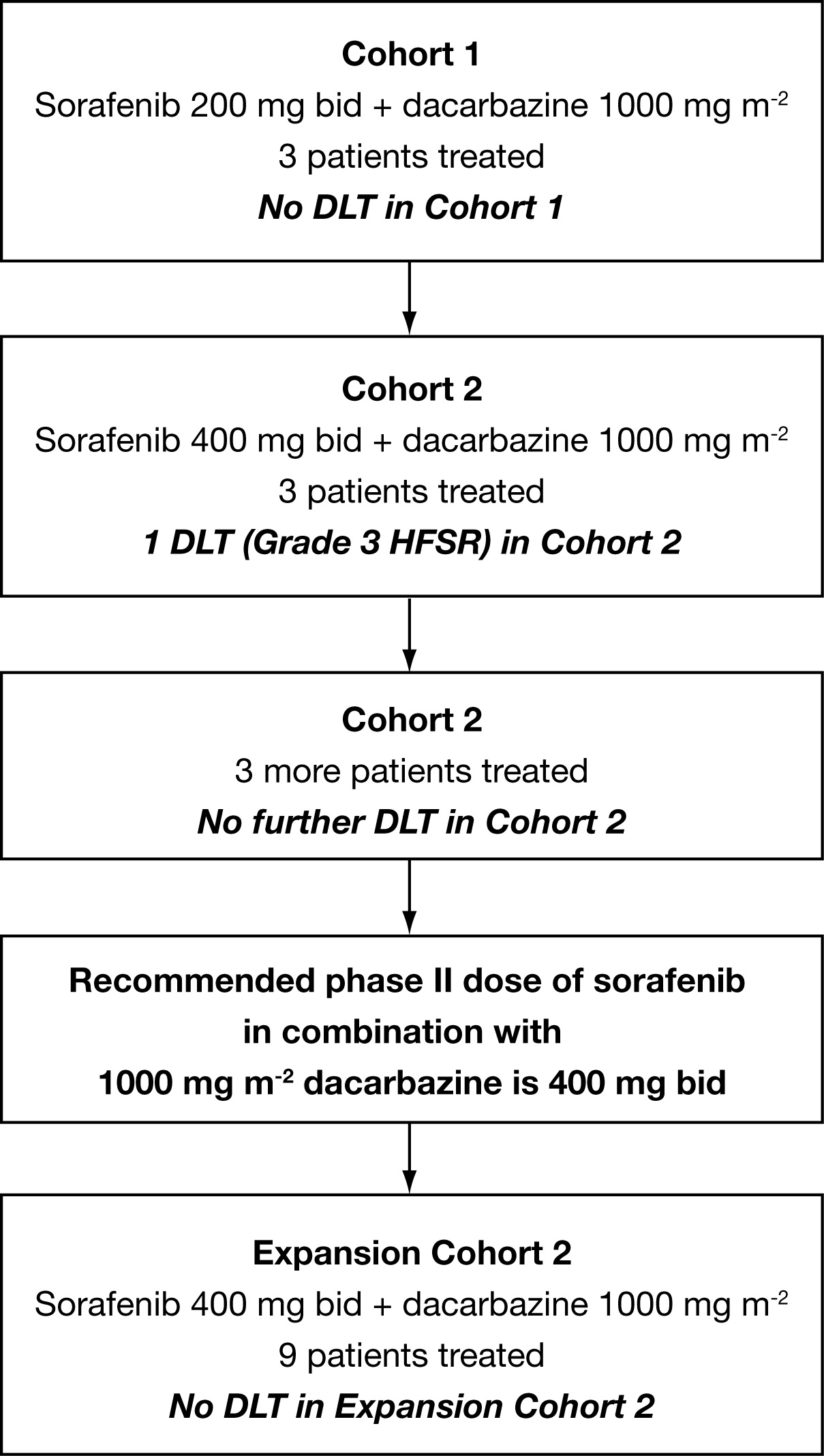
**

**Supplemental Figure 2** Phase II study: largest percentage change from baseline in total tumour length for each subject. Data available from 77 subjects.

**
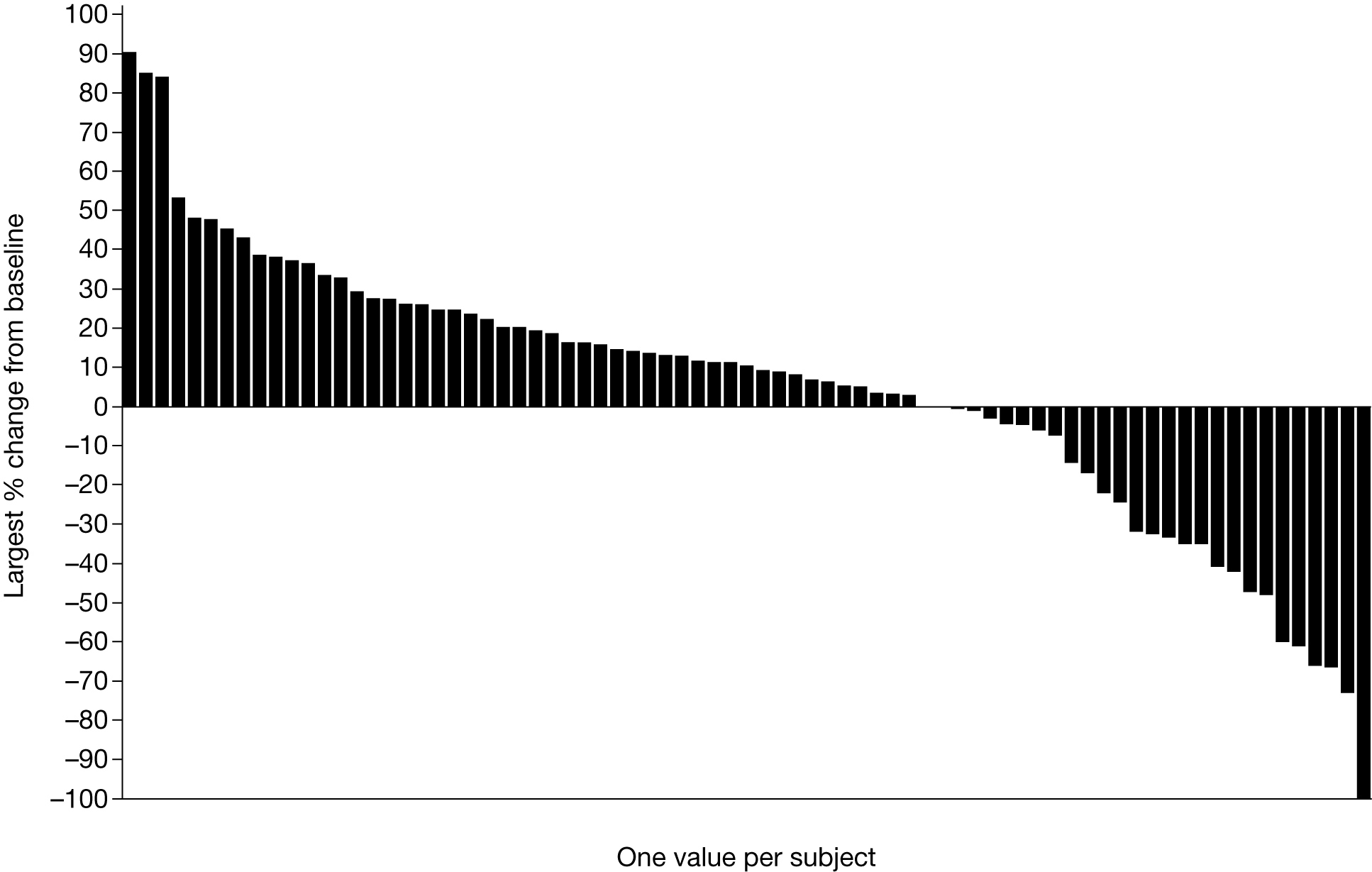
**
